# Geometric priors for reinforcement learning-based multi-robot ecological monitoring

**DOI:** 10.3389/frobt.2026.1934273

**Published:** 2026-09-29

**Authors:** Tamil Selvan Gurunathan, Aryya Gangopadhyay

**Affiliations:** Department of Information Systems, University of Maryland Baltimore County, Baltimore, MD, United States

**Keywords:** geometric priors, Hilbert curves, multi-robot (agent) system, PPO, reinforcement learning, space filling curve

## Abstract

Autonomous ecological monitoring requires robotic systems capable of efficiently exploring large natural environments while minimizing redundant traversal under limited communication and partial environmental knowledge. Although reinforcement learning provides adaptive decision making for multi-robot exploration, sparse rewards often result in inefficient exploration and slow policy convergence. This paper investigates whether locality-preserving geometric priors can improve decentralized reinforcement learning for ecological monitoring. We incorporate Hilbert space-filling curve information through state augmentation, exploration biasing, and potential-based reward shaping while preserving the underlying reinforcement learning algorithms. The framework is implemented using Deep Q-Networks (DQN) and Proximal Policy Optimization (PPO) and evaluated in simulated multi-robot ecological survey environments using teams of 4–16 robots. Performance is evaluated using coverage ratio, redundancy, cumulative reward, convergence speed, and ablation studies. Policies are further validated through outdoor experiments using two Boston Dynamics Spot robots operating in an environment containing natural vegetation and terrain obstacles. Results demonstrate consistent improvements in exploration efficiency, survey coverage, redundancy reduction, and learning performance relative to conventional DQN and PPO, demonstrating the effectiveness of geometric priors for reinforcement learning-based autonomous ecological monitoring.

## 1 Introduction

### 1.1 Motivation

Autonomous coverage and exploration are fundamental capabilities for multi-robot systems operating in complex and partially known environments. A wide range of applications—including environmental monitoring, search and rescue, precision agriculture, habitat monitoring, vegetation mapping, wetland restoration, biodiversity assessment, and invasive species detection—require teams of robots to efficiently traverse large workspaces while minimizing redundant motion and ensuring complete spatial coverage. As the size and complexity of operating environments continue to increase, scalable coverage strategies that can operate with limited communication and decentralized decision making have become increasingly important.

Classical coverage path planning (CPP) algorithms, including boustrophedon decomposition, spiral traversal, and space-filling curve methods, have demonstrated excellent performance in structured and fully known environments for robotic inspection, environmental monitoring, and mapping tasks (Galceran and Carreras, 2013; Huang and Messerschmitt, 2001; Yamauchi et al., 1998). These approaches generate deterministic trajectories that often achieve complete coverage with minimal overlap (Jayalakshmi et al., 2025; Shen et al., 2025a). However, they generally assume that the environment is static and known *a priori*. When obstacles change, new obstacles appear, or environmental uncertainty is introduced, precomputed trajectories may become suboptimal or even infeasible, requiring expensive replanning or centralized coordination.

Reinforcement learning (RL) has emerged as a promising alternative because it enables robots to learn navigation and coverage policies directly from interaction with the environment (Tang et al., 2025) rather than relying solely on handcrafted planning algorithms. In decentralized multi-robot systems, RL offers the potential for adaptive decision making using only local observations, allowing robots to respond to unforeseen environmental changes while avoiding dependence on centralized controllers. Despite these advantages, reinforcement learning for coverage remains challenging. Coverage tasks typically provide sparse and delayed rewards, making efficient exploration difficult during training. Conventional exploration strategies such as random action selection or stochastic policy sampling frequently produce inefficient trajectories characterized by repeated visitation of previously explored regions, slow convergence, and poor sample efficiency, particularly as the number of robots and the size of the environment increase.

One promising direction for addressing these challenges is to incorporate geometric priors into the learning process. Rather than replacing reinforcement learning with predetermined trajectories, geometric priors can provide structured guidance during exploration while allowing the learned policy to retain the flexibility needed to adapt to changing environments. Among these priors, Hilbert space-filling curves possess several desirable properties for robotic coverage. Their locality-preserving mapping transforms two-dimensional workspace coordinates into a one-dimensional traversal sequence while minimizing long-distance transitions and preserving neighborhood relationships. These characteristics suggest that Hilbert curves may provide an effective exploration scaffold, encouraging coherent spatial exploration during learning without constraining the final policy to follow a fixed trajectory.

In this work, we investigate whether integrating Hilbert space-filling curve priors into reinforcement learning can improve exploration efficiency in decentralized multi-robot coverage tasks. Rather than viewing Hilbert curves as complete coverage planners, we treat them as geometric guidance mechanisms that bias early exploration toward locality-preserving trajectories while allowing reinforcement learning to optimize task performance through interaction with the environment. We evaluate this hypothesis through extensive simulation studies and physical robot experiments to determine the extent to which structured exploration can improve coverage efficiency, reduce redundant visitation, and accelerate policy convergence.

Large-scale ecological monitoring requires repeated autonomous coverage of forests, wetlands, grasslands, coastal ecosystems, and restoration sites. Robotic platforms capable of systematically exploring these environments can improve biodiversity assessment, vegetation mapping, soil monitoring, invasive species detection, and post-restoration evaluation while reducing labor-intensive field surveys. These applications require scalable decentralized exploration methods that operate efficiently under limited communication and partial environmental knowledge.

### 1.2 Research gap and objectives

Although reinforcement learning has demonstrated considerable promise for autonomous navigation and multi-robot coverage, efficient exploration remains one of its principal challenges. In coverage tasks, agents often receive sparse or delayed rewards because meaningful feedback is obtained only after substantial portions of the environment have been explored. Consequently, conventional exploration mechanisms, including random action selection, ϵ -greedy exploration, and stochastic policy sampling, frequently generate inefficient trajectories characterized by excessive overlap, repeated visitation of previously explored regions, and slow policy convergence. These limitations become increasingly pronounced as the number of agents and the size of the operating environment increase.

Several approaches have been proposed to improve exploration efficiency (Pathak et al., 2017), curriculum learning (Bengio et al., 2009), active neural SLAM (Chaplot et al., 2020), Metal-RL for Structured Exploration (Gupta et al., 2017), and memory-augmented reinforcement learning (Wayne et al., 2018). However, many of these approaches introduce additional learning objectives, auxiliary neural networks, or complex reward formulations that increase computational cost and training complexity. Moreover, methods that rely on explicit map representations or centralized planning may not be appropriate for decentralized robotic systems operating with limited communication or partial observations (Calzolari et al., 2025; Wakilpoor et al., 2020).

Geometric priors provide an alternative strategy for improving exploration without substantially increasing model complexity. Classical coverage algorithms have long exploited geometric structures, including boustrophedon decomposition, spiral traversal, and space-filling curves, to produce efficient deterministic coverage paths. These methods are computationally efficient and often achieve complete coverage in structured environments. However, they generally function as complete planning algorithms rather than as components of a learning framework. As a result, they typically lack the adaptability required to respond to dynamic environments, unknown obstacles, or changes encountered during execution.

Despite the widespread use of both reinforcement learning and geometric coverage strategies, relatively little attention has been devoted to integrating geometric priors directly into the exploration process of reinforcement learning (Wakode and Sinha, 2023). In particular, it remains unclear whether locality-preserving space-filling curves can serve as effective exploration scaffolds that guide early policy learning while preserving the adaptability of learned behaviors. Furthermore, few studies have systematically investigated the relative contributions of geometric state representations, exploration biasing, and reward shaping (Kliem and Dasgupta, 2025) within a unified reinforcement learning framework.

The objective of this work is to investigate the role of Hilbert space-filling curves as structured exploration priors for decentralized multi-robot coverage. Rather than replacing reinforcement learning with predetermined trajectories, we examine whether geometric guidance can improve learning efficiency while allowing the policy to remain adaptive to environmental changes. Specifically, this study addresses the following research questions:

1. Can locality-preserving Hilbert space-filling curves improve exploration efficiency during reinforcement learning for multi-robot coverage?
2. How do Hilbert-guided policies compare with conventional DQN and PPO approaches in terms of coverage efficiency, redundancy, and convergence speed?
3. What are the relative contributions of Hilbert-based state augmentation, exploration biasing, and reward shaping to the observed performance improvements?
4. Can policies learned using Hilbert-guided exploration be transferred to physical robotic platforms while maintaining reliable execution?

DQN and PPO were selected because they represent two widely adopted reinforcement learning paradigms for robotic navigation: value-based learning and policy-gradient optimization. Evaluating both algorithms allows the proposed framework to be assessed across fundamentally different optimization strategies while isolating the effect of Hilbert-guided exploration. The objective of this work is not to outperform every existing reinforcement learning algorithm, but to demonstrate that locality-preserving geometric priors consistently improve exploration efficiency across representative reinforcement learning paradigms.

### 1.3 Contributions

This study investigates the use of Hilbert space-filling curves as structured exploration priors for reinforcement learning-based multi-robot coverage. Unlike classical coverage path planning algorithms, which generate deterministic traversal paths, the proposed framework incorporates geometric priors into the learning process while allowing the policy to adapt to environmental conditions through reinforcement learning. Rather than replacing learned decision making, Hilbert-guided exploration provides a lightweight mechanism for encouraging coherent spatial exploration during training.

The principal contributions of this work are summarized as follows:

1. A Hilbert-guided structured exploration framework for reinforcement learning. We propose two reinforcement learning frameworks, Hilbert-Augmented Deep Q-Network (H-DQN) and Hilbert-Augmented Proximal Policy Optimization (H-PPO), that incorporate locality-preserving Hilbert space-filling curve information through state augmentation, exploration biasing, and potential-based reward shaping. The proposed approach introduces negligible computational overhead while preserving the adaptability of learned policies.
2. A comprehensive experimental evaluation of structured exploration in decentralized multi-robot coverage. The proposed methods are evaluated across multiple simulated environments using teams ranging from four to sixteen robots. Performance is assessed using coverage ratio, redundancy, cumulative reward, and convergence speed, allowing systematic comparison with conventional DQN and PPO baselines.
3. An ablation analysis of geometric exploration mechanisms. To better understand the contribution of each component, we evaluate the effects of Hilbert-based state augmentation, curve-guided exploration, and reward shaping independently. The ablation experiments provide insight into the relative importance of each mechanism and identify structured exploration as the dominant contributor to the observed performance improvements.
4. Experimental validation on a physical robotic platform. Policies learned in simulation were deployed on two Boston Dynamics Spot quadruped robots operating collaboratively in a representative outdoor ecological monitoring environment using a waypoint interface that converts learned trajectories into executable SE (2) motion commands. The hardware experiments demonstrate that the proposed framework can be transferred from simulation to a physical robot while maintaining reliable trajectory execution and efficient coverage behavior.

The above contributions provide a systematic evaluation of geometric priors as computationally efficient exploration mechanisms for reinforcement learning-based robotic coverage. The results demonstrate that incorporating locality-preserving spatial structure into the exploration process improves learning efficiency and coverage performance while maintaining the flexibility required for decentralized autonomous operation.

## 2 Materials and methods

Figure 1 illustrates the overall reinforcement learning framework investigated in this work. Although the framework is general and independent of any specific application domain, it is evaluated in the context of decentralized ecological monitoring, where efficient exploration of large natural environments is required. The system first maps the robot’s position to a normalized Hilbert index, which is used to augment the local observation and bias exploration during training. The resulting augmented observations are processed by either the H-DQN or H-PPO learning algorithm, and the learned policy is subsequently deployed through the waypoint generation interface for execution on a physical robotic platform.

**FIGURE 1:**
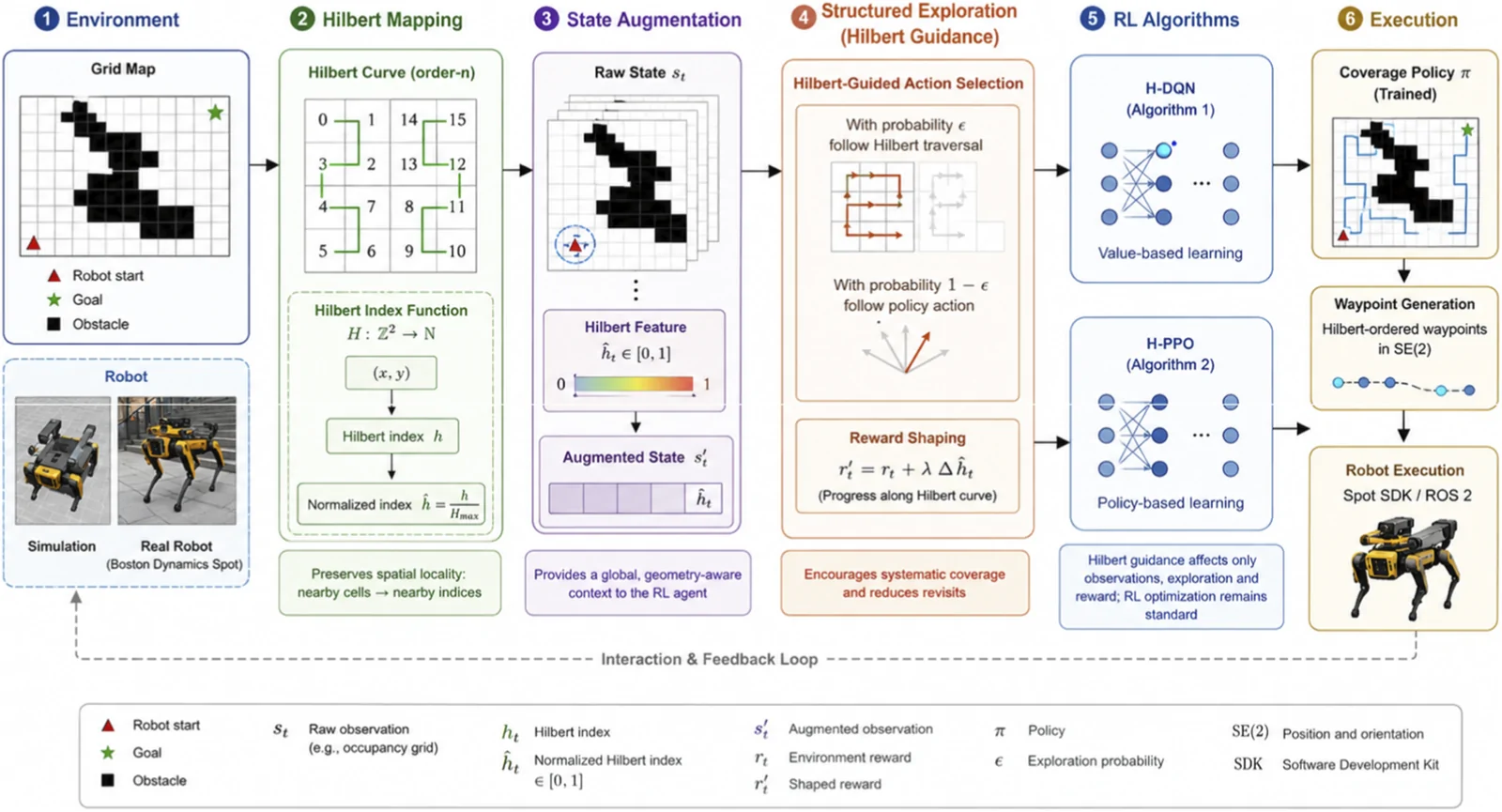
Overview of the proposed architecture.

### 2.1 Coverage problem formulation

We consider the problem of decentralized multi-robot ecological monitoring in a discrete two-dimensional environment represented as a grid map $G\in Z^{M\times N}$ , where each cell corresponds to a survey region within an ecological landscape. Traversable cells represent accessible sampling locations, while occupied cells represent non-traversable terrain features or environmental constraints such as dense vegetation, mature trees, rock outcrops, wetlands, water bodies, steep terrain, or protected habitat zones. This occupancy-grid abstraction provides a controlled and reproducible representation of the spatial constraints commonly encountered during autonomous ecological monitoring missions while remaining independent of any specific ecosystem or sensing modality.

A team of K homogeneous mobile robots is deployed within the environment with the objective of maximizing survey coverage while minimizing redundant revisits to previously explored regions. The proposed framework is independent of the sensing modality and may be integrated with RGB cameras, multispectral or hyperspectral imagers, thermal cameras, LiDAR, or environmental sensing payloads used for applications such as habitat mapping, vegetation surveys, biodiversity assessment, ecosystem restoration, and invasive species monitoring.

Each robot operates independently using only local observations and does not communicate explicitly with other robots during execution. At each decision step t , robot i receives an observation

$$o_{i}^{t}\in O,$$ (1)

consisting of a fixed-size egocentric occupancy map centered on the robot together with its current navigation state. The robot then selects an action

$$a_{i}^{t}\in A,$$ (2)

where the discrete action space is defined as

$$A=\left\{\text{North},\text{South},\text{East},\text{West},\text{Stay}\right\}.$$ (3)

The environment evolves according to the selected actions while respecting terrain constraints, ecological exclusion zones, and workspace boundaries. Following execution of an action, the robot receives a scalar reward together with an updated local observation.

The objective of the coverage task is to maximize the cumulative discounted return

$$J\left(\pi \right)=E\left[\sum_{t=0}^{T}\gamma^{t}r_{t}\right],$$ (4)

where π denotes the control policy, $r_{t}$ is the task reward at time step t , $\left.\gamma \in [0,1\right)$ is the discount factor, and T is the episode horizon.

The reward function encourages systematic ecological surveying by assigning positive rewards when robots visit previously unexplored survey locations, small penalties for revisiting previously observed regions, and collision penalties when attempting to enter non-traversable ecological features or move outside the workspace boundary. Additional Hilbert-guided reward shaping is introduced in Section 2.3.3 to encourage spatially coherent exploration during the early stages of learning.

Unlike centralized coverage approaches, the robots maintain no shared global map and exchange no explicit inter-agent communication during execution. Coordination therefore emerges solely through the interaction between independently learned policies and the shared environment. This decentralized formulation reflects realistic ecological monitoring scenarios in which robotic teams operate over large natural environments where communication bandwidth may be intermittent or unavailable.

### 2.2 Hilbert space-filling curves as geometric priors

Space-filling curves provide continuous mappings between multidimensional spaces and one-dimensional traversal sequences while preserving spatial locality. Among these mappings, the Hilbert space-filling curve (Butz, 1971) has attracted considerable attention in robotics because neighboring locations in the two-dimensional workspace remain close in the corresponding one-dimensional ordering. This locality-preserving property reduces long-distance transitions and naturally produces coherent traversal patterns, making Hilbert curves well suited for robotic coverage, environmental monitoring, and spatial indexing. Previous work has successfully employed space-filling curves for robotic mapping, coverage path planning, and environmental monitoring, demonstrating their effectiveness for systematic workspace traversal (Huang and Messerschmitt, 2001; Shen et al., 2025b; Shen et al., 2025a; Galceran and Carreras, 2013).

Formally, a Hilbert curve defines a mapping

$$H:Z^{2}\to N,$$ (5)

where each grid coordinate $\left(x,y\right)$ is assigned a unique Hilbert index h . Unlike a simple row-wise or column-wise traversal, the recursive construction of the Hilbert curve minimizes discontinuities by ensuring that consecutive indices correspond to spatially adjacent cells whenever possible. Consequently, the resulting traversal exhibits strong locality preservation across multiple spatial scales.

The locality-preserving characteristic of Hilbert curves offers several desirable properties for reinforcement learning-based coverage. First, nearby states in the physical environment remain nearby in the Hilbert ordering, encouraging smooth transitions during exploration and reducing unnecessary long-distance movements. Second, the recursive construction provides a globally consistent traversal sequence that can be computed efficiently using integer arithmetic, introducing negligible computational overhead during both training and deployment. Third, because the Hilbert index is represented as a single scalar value, it provides a compact spatial descriptor that can be incorporated into reinforcement learning policies without substantially increasing the dimensionality of the observation space.

It is important to emphasize that the Hilbert curve is not used in this work as a deterministic coverage planner. Classical coverage path planning algorithms generate complete traversal paths that agents are expected to follow during execution. In contrast, the proposed framework employs the Hilbert curve only as a geometric prior that influences the exploration process during reinforcement learning. The learned policy remains free to deviate from the Hilbert ordering whenever environmental conditions or accumulated experience indicate that alternative actions produce higher long-term reward. Consequently, the geometric prior guides early exploration while preserving the adaptability that characterizes reinforcement learning.

Alternative spatial representations, including Cartesian coordinates, Morton (Z-order) curves, and row-major traversals, can also provide positional information to learning agents. Morton (Z-order) curves provide a computationally efficient alternative, although Hilbert curves generally preserve spatial locality more effectively for coverage applications (Butz, 1971). However, Hilbert curves exhibit stronger locality-preserving behavior than many of these alternatives, particularly with respect to minimizing long-range discontinuities during traversal. By maintaining neighborhood relationships throughout the recursive mapping, Hilbert curves provide an exploration scaffold that encourages coherent spatial progression without imposing rigid movement constraints. This property motivates their use as structured exploration priors in the present study.

Figure 2 illustrates a fourth-order Hilbert space-filling curve together with the corresponding traversal sequence used to compute the normalized Hilbert indices employed throughout this work. During reinforcement learning, the Hilbert index serves two complementary purposes: (1) as an additional feature within the agent’s state representation and (2) as a reference trajectory for structured exploration during the early stages of policy learning. These two mechanisms are evaluated independently through the ablation studies presented in Section 3.

**FIGURE 2:**
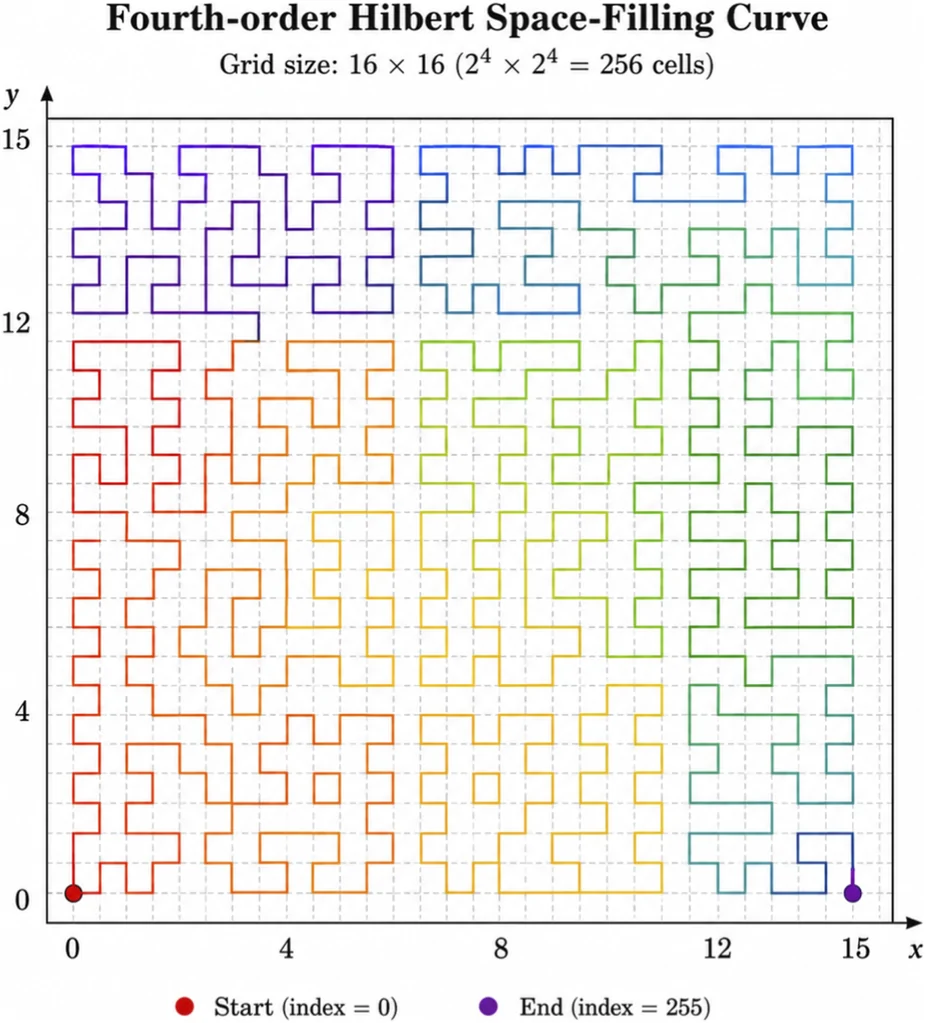
Fourth-order Hilbert space-filling curve illustrating the recursive traversal of a 16×16 grid (256 cells). The traversal begins at index 0 (red) and terminates at index 255 (purple). The Hilbert ordering preserves spatial locality by mapping neighboring cells in the two-dimensional workspace to nearby indices in the one-dimensional traversal sequence. During the experiments, fifth-order ( 32×32 ) and sixth-order ( 64×64 ) Hilbert curves were generated to match the corresponding simulation environments.

### 2.3 Hilbert-guided structured exploration

The central idea of the proposed framework is to use a Hilbert space-filling curve as a geometric prior that guides exploration during reinforcement learning. Rather than prescribing a deterministic coverage path, the Hilbert curve provides a structured spatial reference that encourages coherent exploration while preserving the adaptability of the learned policy. Consequently, the proposed framework combines the systematic traversal properties of classical coverage algorithms with the decision-making flexibility of reinforcement learning. The proposed framework shares the objective of improving exploration efficiency with previous work on frontier-based exploration, curiosity-driven learning, active neural mapping, and systematic exploration strategies (Yamauchi, 1997; Pathak et al., 2017; Chaplot et al., 2020; Ecoffet et al., 2021; Yu et al., 2023; Chiun et al., 2025). The proposed exploration strategy in this paper consists of three complementary components:

1. Hilbert-based state augmentation,
2. Hilbert-guided exploration bias, and
3. Hilbert-based reward shaping.

Each component contributes differently to the learning process and is evaluated independently through the ablation studies presented in Section 3.

#### 2.3.1 Hilbert-based state augmentation

For each robot, the current grid position is converted into a normalized Hilbert index using the mapping described in Section 2.2. Let

$$h_{t}=\frac{H\left(x_{t},y_{t}\right)}{H_{\max}},$$ (6)

where $H\left(x_{t},y_{t}\right)$ denotes the Hilbert index of the robot’s current location and $H_{\max}$ is the maximum index for the environment.

The normalized Hilbert index is concatenated with the robot’s local observation to produce the augmented state

$$s_{t}^{\prime }=\left[s_{t},h_{t}\right].$$ (7)

The additional scalar introduces global spatial context while increasing the observation dimensionality by only one feature.

Unlike explicit map sharing or centralized planning, the Hilbert index does not encode the positions or intentions of neighboring robots. Instead, it provides each agent with a compact representation of its relative location within the global traversal sequence.

#### 2.3.2 Hilbert-guided exploration bias

Standard reinforcement learning algorithms typically rely on stochastic action selection during training to encourage exploration. While effective in many domains, purely random exploration often produces fragmented trajectories with excessive revisitation in large coverage environments.

To encourage spatially coherent exploration, the proposed framework introduces a probabilistic exploration bias during training. With probability ϵ , the robot selects the action that advances it toward the next reachable Hilbert cell. Otherwise, the action is selected according to the underlying reinforcement learning policy,

$$a_{t}=\left\{\begin{array}{ll} a_{H}, & \text{with probability }\epsilon ,\\ a_{\pi }, & \text{otherwise}, \end{array}\right.$$ (8)

where $a_{H}$ denotes the Hilbert-guided action and $a_{\pi }$ represents the action generated by the reinforcement learning policy.

The exploration probability is gradually annealed throughout training,

$$\epsilon_{k+1}=\max\left(\epsilon_{\min},\kappa \epsilon_{k}\right),$$ (9)

where κ<1 is the decay factor.

Consequently, the influence of the geometric prior decreases as training progresses, allowing the learned policy to dominate decision making during later stages of optimization.

#### 2.3.3 Hilbert-based reward shaping

In sparse-reward environments, informative feedback may occur only after large portions of the environment have been explored. To accelerate learning, the proposed framework incorporates potential-based reward shaping.

The modified reward is defined as

$$r_{t}^{\prime }=r_{t}+\gamma \Phi \left(h_{t+1}\right)-\Phi \left(h_{t}\right),$$ (10)

where $r_{t}$ denotes the original task reward, γ is the discount factor defined in Equation 4, and $\Phi \left(\cdot \right)$ is the potential function used for reward shaping, and

$$\Phi \left(h_{t}\right)=\alpha h_{t},$$ (11)

where α controls the influence of the shaping function.

This formulation preserves the optimal policy under the original Markov Decision Process while encouraging progression along locality-preserving traversal sequences during the early stages of learning.

#### 2.3.4 Overall learning framework

The proposed framework is intentionally independent of any specific reinforcement learning algorithm. Hilbert-guided structured exploration modifies only the exploration process and state representation while leaving the underlying optimization algorithm unchanged. Consequently, the framework can be integrated with value-based, policy-gradient, or actor-critic reinforcement learning methods with minimal modification.

In this work, the framework is implemented using both Deep Q-Networks (H-DQN) and Proximal Policy Optimization (H-PPO), described in Sections 2.4, 2.5, respectively. The corresponding experimental evaluation investigates the contribution of each component to coverage efficiency, redundancy reduction, and convergence speed.

### 2.4 Hilbert-Augmented Deep Q-Network

To evaluate the generality of the proposed structured exploration framework, we first integrate it with the Deep Q-Network (DQN) algorithm. The proposed framework builds upon the original Deep Q-Network algorithm introduced by Mnih et al. (2015), while modifying only the observation representation and exploration strategy. DQN approximates the optimal action-value function using a deep neural network that estimates the expected long-term return associated with each discrete action. The network parameters are optimized by minimizing the temporal-difference (TD) error while employing experience replay and a periodically updated target network to stabilize learning.

In the proposed Hilbert-Augmented Deep Q-Network (H-DQN), the underlying DQN optimization procedure remains unchanged. Instead, the structured exploration framework modifies the learning process through three mechanisms introduced in Section 2.3:

1. Hilbert-based state augmentation,
2. Hilbert-guided exploration bias, and
3. Hilbert-based reward shaping.

The augmented observation presented to the Q-network is defined as

$$s_{t}^{\prime }=\left[s_{t},h_{t}\right],$$ (12)

where $s_{t}$ denotes the original local observation and $h_{t}$ is the normalized Hilbert index corresponding to the robot’s current grid location.

The Q-network therefore learns an action-value function

$$Q\left(s_{t}^{\prime },a_{t};\theta \right),$$ (13)

where θ represents the trainable network parameters.

During training, exploration follows the structured policy described in Section 2.3. With probability ϵ , the agent selects the Hilbert-guided action that advances exploration toward the next reachable Hilbert cell. Otherwise, the action is selected according to the conventional ϵ -greedy DQN policy.

The target value used during temporal-difference learning is

$$y_{t}=r_{t}^{\prime }+\gamma \max_{a^{\prime }}Q\left(s_{t+1}^{\prime },a^{\prime };\theta^{-}\right),$$ (14)

where $\theta^{-}$ denotes the parameters of the target network and $r_{t}^{\prime }$ is the shaped reward defined in Equation 10.

The DQN loss function remains identical to the conventional formulation,

$$L\left(\theta \right)=E\left[{\left(y_{t}-Q\left(s_{t}^{\prime },a_{t};\theta \right)\right)}^{2}\right].$$ (15)

Consequently, the proposed framework does not alter the optimization objective of DQN. Instead, it modifies the trajectories from which experience is collected, encouraging the replay buffer to contain more coherent and informative coverage trajectories during the early stages of training.

Algorithm 1 summarizes the complete H-DQN training procedure. At each interaction step, the robot first computes its normalized Hilbert index and augments the local observation. Structured exploration is then performed according to the exploration schedule described in Section 2.3. The resulting transition is stored in the replay buffer, after which minibatches are sampled for standard DQN optimization. Periodic synchronization of the target network is performed exactly as in the original DQN algorithm.

Algorithm 1 Hilbert-Augmented Deep Q-Network (H-DQN).

   1:  Initialize Q-network $Q\left(s,a;\theta \right)$

   2:  Initialize target network $Q^{\prime }\left(s,a;\theta^{-}\right)$

   3:  Initialize replay buffer D

   4:  **for** each training episode **do**
   5:   Observe current state $s_{t}$

   6:   Compute Hilbert index $h_{t}$

   7:   Form augmented state $s_{t}^{\prime }=\left[s_{t},h_{t}\right]$

   8:   **if** random() < ϵ **then**
   9:    Select Hilbert-guided action $a_{H}$

10:   **else**
11:    Select action using ϵ -greedy DQN
12:   **end if**
13:   Execute action and observe $\left(r_{t},s_{t+1}\right)$

14:   Compute normalized Hilbert index $h_{t+1}$

15:   Store $\left(s_{t}^{\prime },a_{t},r_{t}^{\prime },s_{t+1}^{\prime }\right)$ in replay buffer
16:   Sample mini-batch
17:   Update Q-network using the standard DQN loss
18:   Periodically update target network
19:  **end for**

The proposed modifications are confined to the observation representation and exploration process, allowing H-DQN to retain the computational complexity of the original algorithm. Hilbert index computation requires only simple integer arithmetic and introduces negligible overhead relative to neural network inference and optimization. Consequently, the framework can be integrated into existing DQN implementations with minimal modification while remaining fully compatible with standard reinforcement learning software libraries.

### 2.5 Hilbert-augmented Proximal Policy Optimization

To investigate the applicability of the proposed structured exploration framework to policy-gradient methods, we integrate Hilbert-guided exploration with Proximal Policy Optimization (PPO). PPO is a widely used actor–critic reinforcement learning algorithm that updates stochastic policies using a clipped surrogate objective designed to improve training stability while avoiding excessively large policy updates. The policy optimization procedure follows the clipped surrogate objective proposed by Schulman et al. (2017), with Hilbert-guided exploration influencing only rollout generation.

As with H-DQN, the proposed framework does not modify the underlying optimization procedure of PPO. Instead, the structured exploration framework influences the trajectories collected during training through Hilbert-based state augmentation, exploration biasing, and reward shaping while leaving the policy optimization objective unchanged.

The policy network receives the augmented observation

$$s_{t}^{\prime }=\left[s_{t},h_{t}\right],$$ (16)

where $s_{t}$ denotes the original local observation and $h_{t}$ is the normalized Hilbert index described in Section 2.2.

The stochastic policy therefore becomes

$$\pi_{\theta }\left(a_{t}|s_{t}^{\prime }\right),$$ (17)

where θ denotes the trainable policy parameters.

During rollout collection, actions are generated using the structured exploration mechanism introduced in Section 2.3. With probability ϵ , the robot executes the Hilbert-guided action that advances exploration toward the next reachable Hilbert cell. Otherwise, actions are sampled directly from the stochastic policy. The exploration probability is gradually annealed throughout training, allowing the influence of the geometric prior to decrease as the learned policy improves.

Policy optimization is performed using the conventional PPO clipped surrogate objective

$$L^{\text{CLIP}}\left(\theta \right)=E\left[\min\left(r_{t}\left(\theta \right){\hat{A}}_{t},\text{clip}\left(r_{t}\left(\theta \right),1-\epsilon_{c},1+\epsilon_{c}\right){\hat{A}}_{t}\right)\right],$$

where

$$r_{t}\left(\theta \right)=\frac{\pi_{\theta }\left(a_{t}|s_{t}^{\prime }\right)}{\pi_{\theta_{\text{old}}}\left(a_{t}|s_{t}^{\prime }\right)},$$ (18)

and ${\hat{A}}_{t}$ denotes the generalized advantage estimate (GAE). Throughout this work, GAE is computed using λ=0.95 , and policy updates employ the standard clipped PPO formulation without modification.

Unlike conventional PPO, which relies solely on stochastic policy sampling during rollout collection, the proposed framework introduces a structured exploration mechanism during the early stages of learning. The purpose of this mechanism is not to replace the learned policy but rather to encourage the collection of more informative trajectories in sparse-reward coverage environments. As the exploration probability is annealed toward its minimum value, trajectory generation becomes increasingly dominated by the learned policy itself.

Algorithm 2 summarizes the complete H-PPO training procedure. During each interaction step, the robot computes its normalized Hilbert index, augments the local observation, and performs structured exploration according to the exploration schedule described in Section 2.3. Rollouts are subsequently used to compute generalized advantage estimates and update both the policy and value networks using the standard PPO optimization procedure.

Algorithm 2 Hilbert-Augmented Proximal Policy Optimization (H-PPO).

   1:  Initialize policy $\pi_{\theta }$

   2:  Initialize value function $V_{\phi }$

   3:  **for** each training iteration **do**
   4:   **for** each rollout step **do**
   5:    Observe state $s_{t}$

   6:    Compute Hilbert index $h_{t}$

   7:    Form augmented state $s_{t}^{\prime }=\left[s_{t},h_{t}\right]$

   8:    **if** random() < ϵ **then**
   9:     Execute Hilbert-guided action
10:    **else**
11:     Sample action from $\pi_{\theta }\left(a|s_{t}^{\prime }\right)$

12:    **end if**
13:    Store transition
14:   **end for**
15:   Compute generalized advantage estimates
16:   Update policy using the standard PPO clipped objective
17:   Update value network
18:   Anneal exploration probability ϵ

19:  **end for**

Because the proposed framework modifies only the observation representation and exploration strategy, it introduces negligible computational overhead beyond conventional PPO. Hilbert index computation requires only integer arithmetic and a single additional scalar input to the policy network. Consequently, the proposed method preserves the computational efficiency of PPO while providing a lightweight geometric prior that improves exploration in decentralized coverage tasks.

#### 2.5.1 Computational complexity

The proposed framework preserves the computational complexity of the underlying reinforcement learning algorithms because it modifies only the observation representation, exploration strategy, and reward computation. Hilbert index generation requires only integer arithmetic and is performed once per decision step, resulting in constant-time complexity, $O\left(1\right)$ , with constant additional memory. State augmentation adds a single normalized scalar to the observation vector, while exploration guidance requires only a comparison between the current and neighboring Hilbert indices. Consequently, the dominant computational cost remains neural network inference and optimization, which are identical to those of the original DQN and PPO implementations. In practice, the proposed framework introduces negligible computational and memory overhead while remaining fully compatible with existing reinforcement learning software libraries. A summary of the theoretical time complexity and memory overhead introduced by each framework component is provided in Table 1.

**TABLE 1:** Additional computational overhead introduced by the proposed framework.

Component	Time complexity	Memory overhead
Hilbert index computation	$O\left(1\right)$	$O\left(1\right)$
State augmentation	$O\left(1\right)$	+1 scalar feature
Exploration guidance	$O\left(1\right)$	None
Reward shaping	$O\left(1\right)$	None
Neural network inference	Unchanged	Unchanged
Policy optimization	Unchanged	Unchanged

### 2.6 Experimental platform and hardware implementation

The proposed framework was evaluated through both simulation and physical robot experiments to assess its effectiveness for decentralized ecological monitoring tasks and its feasibility for deployment on autonomous robotic platforms. The experimental environments were intentionally designed as controlled coverage benchmarks that isolate the effects of the proposed exploration strategy while representing the fundamental spatial challenges encountered during ecological monitoring missions. These include systematic exploration of large survey regions containing natural terrain constraints, limited prior environmental knowledge, and sparse opportunities for acquiring scientifically meaningful observations.

#### 2.6.1 Simulation environment

Simulation experiments were conducted in discrete two-dimensional occupancy-grid environments representing ecological survey regions. Two workspace sizes, 32×32 and 64×64 cells, were considered to evaluate scalability across landscapes of increasing spatial complexity. Traversable cells represent accessible survey locations, whereas occupied cells represent non-traversable ecological terrain features such as dense vegetation, mature trees, rock outcrops, wetlands, streams, or protected habitat zones. Although these terrain features were generated procedurally, they capture the fragmented spatial structure commonly encountered during ecological monitoring and ecosystem restoration activities while maintaining reproducible benchmark environments for algorithm evaluation.

During each episode, teams consisting of 4, 8, 12, or 16 homogeneous mobile robots were initialized at predefined starting locations and tasked with systematically surveying the environment while maximizing coverage and minimizing redundant observations.

Each robot received a local 5×5 egocentric occupancy map together with its current navigation state. The action space consisted of five discrete actions corresponding to movement toward the four cardinal directions or remaining stationary. Episodes terminated when either the maximum number of time steps was reached or all accessible survey locations had been visited.

#### 2.6.2 Learning configuration

All reinforcement learning models were implemented using PyTorch and trained using the Adam optimizer with a learning rate of $3\times 10^{-4}$ . For PPO, the clipping parameter was set to 0.2, while generalized advantage estimation employed λ=0.95 . DQN utilized an experience replay buffer together with periodically updated target networks. All experiments were trained for 300,000 interaction steps using a batch size of 64.

The Hilbert-guided exploration probability was initialized to ϵ=0.30 and gradually annealed to ϵ=0.05 throughout training. Hilbert indices were normalized to the interval $\left[0,1\right]$ before being incorporated into the observation vector.

#### 2.6.3 Physical robot implementation

To evaluate deployment feasibility, policies learned entirely in simulation were executed on two Boston Dynamics Spot quadruped robots. The physical experiments were conducted in a natural field site in which autonomous robots systematically traverse predefined survey regions while avoiding environmental constraints.

Policy-generated survey trajectories were converted into executable planar trajectories through a waypoint interface developed using the Boston Dynamics Spot Software Development Kit (SDK). Each waypoint consisted of a planar pose

$$\left(x,y,\theta \right)\in SE\left(2\right),$$ (19)

where $\left(x,y\right)$ denotes the robot position and θ its heading.

Waypoint trajectories were transformed into Spot-compatible velocity and pose commands while maintaining lease management, time synchronization, emergency stop protection, and obstacle safety constraints provided by the SDK. Motion execution employed discrete heading changes together with forward translation commands to generate smooth survey trajectories suitable for deployment on the quadruped platform.

Trajectory execution was performed within the VISION coordinate frame using curvature-constrained velocity profiles to ensure dynamically feasible motion. Obstacle inflation and keep-out regions were incorporated into the generated trajectories to improve execution safety and reduce localization failures.

The hardware implementation serves as a proof of decentralized multi-robot deployment rather than a field validation of ecological monitoring. Its primary objective is to demonstrate that policies learned using Hilbert-guided structured exploration can be transferred from simulation to a physical robotic platform and executed reliably. The framework is intended to be integrated with application-specific sensor payloads, including RGB cameras, multispectral imagers, thermal cameras, LiDAR, hyperspectral sensors, and environmental sensing systems, for future ecological monitoring and ecosystem restoration missions.

### 2.7 Experimental design

The proposed framework was evaluated through a series of simulation and hardware experiments designed to assess exploration efficiency, survey coverage, scalability, learning performance, and deployment feasibility for autonomous ecological monitoring. All experiments employed identical training protocols, neural network architectures, and evaluation procedures to ensure fair comparison between conventional reinforcement learning algorithms and their Hilbert-augmented counterparts.

Although the experimental evaluation was conducted using controlled occupancy grid benchmark environments, these environments capture the fundamental spatial exploration problems encountered during ecological monitoring. Applications such as habitat mapping, biodiversity assessment, vegetation surveys, wetland monitoring, ecosystem restoration, and invasive species detection all require autonomous robots to systematically explore large natural environments while minimizing redundant observations and operating under limited communication. The occupancy-grid abstraction therefore provides a reproducible benchmark for evaluating exploration strategies independently of any specific ecosystem or sensing modality, allowing the underlying reinforcement learning methodology to be isolated from application-specific perception challenges.

#### 2.7.1 Simulation experiments

Simulation studies were performed using discrete two-dimensional occupancy grids of size 32×32 and 64×64 cells representing ecological survey regions. Each environment consisted of accessible survey locations together with procedurally generated terrain constraints representing features such as dense vegetation, mature trees, wetlands, streams, rock outcrops, and protected habitat zones while preserving overall workspace connectivity.

For scalability experiments, homogeneous robot teams consisting of 4, 8, 12, and 16 agents were evaluated under identical environmental conditions. Each robot received a local 5×5 egocentric observation augmented with the normalized Hilbert index described in Section 2.3. Robots selected one of five discrete actions corresponding to movement toward the four cardinal directions or remaining stationary. Episodes terminated when all accessible survey locations had been visited or a predefined maximum number of interaction steps had been reached.

Both DQN and PPO models were trained for 300,000 interaction steps using identical hyperparameter settings and random initialization seeds. Five independent training runs were performed for every experimental configuration using random seeds $\left\{42,97,123,888,2025\right\}$ , allowing statistical evaluation of performance variability and reproducibility.

#### 2.7.2 Baseline methods

The proposed Hilbert-augmented reinforcement learning algorithms were compared against conventional reinforcement learning baselines using identical neural network architectures, optimizer settings, and training protocols. The evaluated methods consisted of

- Standard Deep Q-Network (DQN),
- Hilbert-Augmented Deep Q-Network (H-DQN),
- Standard Proximal Policy Optimization (PPO), and
- Hilbert-Augmented Proximal Policy Optimization (H-PPO).

For each algorithm, all hyperparameters, optimizer settings, neural network architectures, training duration, and evaluation procedures were held constant. The only differences between the baseline and proposed methods were the Hilbert-based state augmentation, exploration bias, and reward-shaping mechanisms introduced in Section 2.3. This design isolates the contribution of the proposed geometric priors while ensuring a fair comparison across all evaluated reinforcement learning algorithms.

Recent exploration methods such as curiosity-driven learning, active neural mapping, frontier-based exploration, MAPPO, and QMIX address exploration using different mechanisms, including intrinsic rewards, centralized training, or auxiliary objectives. These approaches are complementary to the proposed framework. DQN and PPO were selected because they provide representative value-based and policy-gradient baselines while allowing the contribution of the proposed geometric prior to be isolated.

#### 2.7.3 Ablation study

To quantify the contribution of each component of the proposed framework, three ablation experiments were conducted using the H-PPO implementation:

1. Removal of Hilbert-based state augmentation,
2. Removal of Hilbert-guided exploration bias, and
3. Removal of Hilbert-based reward shaping.

Each ablated model was trained using the same network architecture, hyperparameters, random initialization seeds, and training duration as the complete H-PPO framework. Performance differences were subsequently evaluated using the metrics defined in Section 2.8 to determine the relative contribution of each component to ecological survey efficiency, coverage quality, learning performance, and exploration behavior.

### 2.8 Performance metrics

The proposed framework was evaluated using quantitative metrics that characterize exploration efficiency, coverage quality, learning performance, and deployment feasibility. These metrics were computed consistently across all simulation experiments, while path fidelity and execution time were additionally evaluated during hardware deployment.

#### 2.8.1 Coverage ratio

Coverage ratio measures the proportion of traversable cells visited at least once during an episode. It is defined as

$$C=\frac{N_{\text{visited}}}{N_{\text{free}}},$$ (20)

where $N_{\text{visited}}$ denotes the number of unique free cells visited by one or more robots and $N_{\text{free}}$ represents the total number of traversable cells in the environment.

Coverage ratio ranges from 0 to 1, with larger values indicating more complete exploration.

#### 2.8.2 Coverage redundancy

Efficient coverage requires minimizing repeated visits to previously explored locations. Redundancy is therefore computed as

$$R=\frac{N_{\text{visits}}}{N_{\text{visited}}},$$ (21)

where $N_{\text{visits}}$ is the total number of cell visits accumulated by all robots throughout an episode.

Lower redundancy values indicate more efficient exploration with reduced overlap among robot trajectories.

#### 2.8.3 Cumulative reward

Learning performance is evaluated using the cumulative discounted reward accumulated during each episode,

$$G=\sum_{t=0}^{T}\gamma^{t}r_{t},$$ (22)

where $r_{t}$ denotes the reward received at time step t , γ is the discount factor, and T is the episode length.

Average cumulative reward provides an overall measure of policy quality throughout the learning process.

#### 2.8.4 Convergence speed

To quantify learning efficiency, convergence speed is measured using a stability-based criterion. Let

$${\bar{G}}_{w}=\frac{1}{w}\sum_{i=t-w+1}^{t}G_{i}$$ (23)

denote the rolling average reward over a window of w evaluation episodes.

Training is considered converged when the rolling average remains above 90% of the maximum observed reward for ten consecutive evaluation windows. The corresponding convergence episode is denoted by

$$T_{\text{conv}}.$$ (24)

This criterion provides a more robust estimate of convergence than isolated reward peaks that may arise from stochastic exploration.

#### 2.8.5 Sample efficiency

Sample efficiency is quantified by the rate at which the reinforcement learning algorithm approaches stable performance. In addition to the convergence episode, reward curves are evaluated throughout training to compare the amount of interaction required for each method to achieve comparable performance levels.

Methods requiring fewer interaction steps to reach stable policies are considered more sample efficient.

#### 2.8.6 Path fidelity

For physical experiments, path fidelity evaluates the agreement between the planned trajectory generated by the reinforcement learning policy and the trajectory executed by the physical robot.

Let

$$P=\left\{p_{1},p_{2},\ldots ,p_{n}\right\}$$ (25)

represent the planned trajectory and

$$E=\left\{e_{1},e_{2},\ldots ,e_{n}\right\}$$ (26)

the corresponding executed trajectory estimated from onboard odometry.

The mean lateral tracking error is computed as

$$F=\frac{1}{n}\sum_{i=1}^{n}∥p_{i}-e_{i}∥,$$ (27)

where smaller values indicate closer agreement between planned and executed motion.

#### 2.8.7 Coverage time

For physical robot experiments, total coverage time is measured as the elapsed time between the robot’s initial motion and the first instant at which every reachable grid cell has been visited.

Coverage time provides a practical measure of deployment efficiency and complements the simulation-based learning metrics by evaluating real-world execution performance.

Collectively, these metrics characterize both learning behavior and operational performance. Coverage ratio and redundancy quantify exploration quality, cumulative reward and convergence speed measure learning efficiency, while path fidelity and coverage time assess the transferability of learned policies to physical robotic platforms.

### 2.9 Statistical analysis

To evaluate the reproducibility and statistical significance of the proposed framework, all simulation experiments were performed using five independent training runs with different random initialization seeds. Unless otherwise stated, all reported performance values correspond to the arithmetic mean across the five experimental repetitions.

Variability in performance was quantified using the sample mean

$$\bar{x}=\frac{1}{n}\sum_{i=1}^{n}x_{i},$$ (28)

and the sample standard deviation

$$s=\sqrt{\frac{1}{n-1}\sum_{i=1}^{n}{\left(x_{i}-\bar{x}\right)}^{2}},$$ (29)

where n=5 represents the number of independent experimental runs.

For each evaluation metric, results are reported as

$$\bar{x}\pm s,$$ (30)

together with corresponding 95% confidence intervals computed as

$$\bar{x}\pm t_{0.975,n-1}\frac{s}{\sqrt{n}},$$ (31)

where $t_{0.975,n-1}$ denotes the critical value of Student’s t distribution for n−1 degrees of freedom.

Statistical comparisons between baseline reinforcement learning methods and their Hilbert-augmented counterparts were performed using Welch’s two-sample t -test, which does not assume equal population variances. Differences were considered statistically significant when

p<0.05. (32)

For experiments involving multiple robot team sizes, performance statistics were computed independently for each team size. Likewise, ablation experiments were analyzed independently to quantify the contribution of each component of the proposed framework.

Learning curves are presented as the mean performance across independent runs, while shaded regions represent one standard deviation. This visualization provides an estimate of training variability and enables qualitative comparison of convergence behavior between competing methods.

The hardware experiments consisted of five repeated executions of each trained policy under identical environmental conditions. Hardware performance metrics, including coverage time, redundancy, and path fidelity, are reported using the same descriptive statistics. Because of the limited number of physical trials, the hardware experiments are intended primarily to demonstrate deployment feasibility rather than to establish statistically significant performance differences.

All statistical analyses were performed using standard numerical computing libraries implemented in Python. The complete experimental configuration, random seeds, evaluation scripts, and raw experimental logs are provided with the supplementary material to facilitate independent verification and reproduction of the reported results. The statistical reporting follows common recommendations for empirical machine learning and robotics studies, including multiple independent training runs, confidence intervals, and hypothesis testing. (https://github.com/TheNameisTamil/H_PPO-H_DQN.git).

## 3 Results

Figure 3 compares representative coverage trajectories generated by the baseline PPO policy and the proposed H-PPO framework under identical environmental conditions.

**FIGURE 3:**
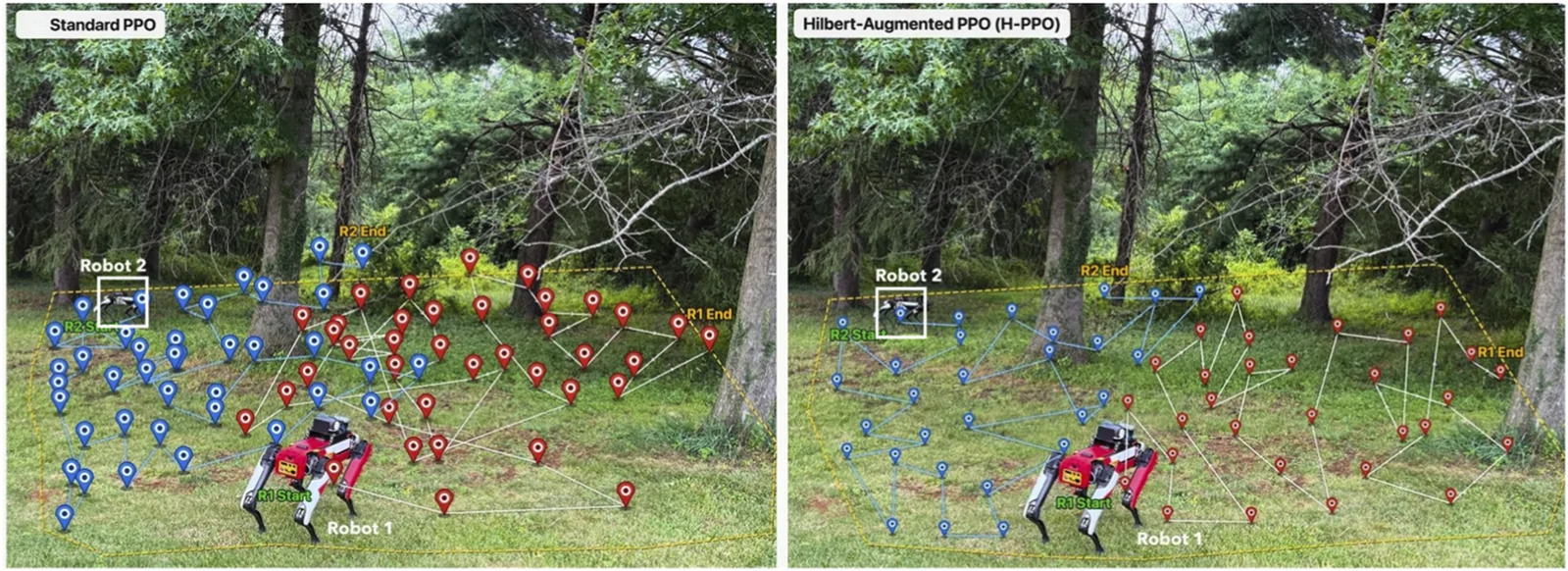
Representative outdoor ecological monitoring experiment using two Boston Dynamics Spot robots. The colored trajectories illustrate the exploration paths executed by both robots under identical environmental conditions generated by (left) the standard PPO policy and (right) the proposed H-PPO policy under identical environmental conditions. H-PPO produces a more coherent locality-preserving traversal with fewer redundant revisits and more complete coverage than the baseline PPO implementation.

### 3.1 Overall coverage performance

The primary objective of this study was to determine whether Hilbert-guided structured exploration improves multi-robot coverage performance relative to conventional reinforcement learning methods. Coverage performance was evaluated using the coverage ratio and redundancy metrics defined in Section 2.8. Results were averaged over five independent training runs for each experimental configuration.

Table 2 summarizes the average coverage ratio achieved by DQN, H-DQN, PPO, and H-PPO for robot teams consisting of 4, 8, 12, and 16 agents. Across all team sizes, both Hilbert-augmented methods achieved higher coverage ratios than their corresponding baseline algorithms. The largest improvements were observed for H-PPO, which consistently achieved the highest average coverage among the evaluated reinforcement learning methods.

**TABLE 2:** Average coverage ratio across agent team sizes.

Method	4 Agents	8 Agents	12 Agents	16 Agents
DQN	0.676	0.729	0.721	0.589
H-DQN (ours)	**0.831**	**0.882**	**0.890**	**0.895**
PPO	0.712	0.760	0.772	0.683
H-PPO (ours)	**0.871**	**0.904**	**0.917**	**0.927**

For example, with sixteen robots, the average coverage ratio increased from 0.589 for DQN to 0.895 for H-DQN and from 0.683 for PPO to 0.927 for H-PPO. Similar improvements were observed across all smaller team sizes, indicating that the proposed structured exploration framework consistently increased the proportion of the environment visited during each episode.

Table 3 presents the corresponding redundancy measurements. In contrast to the baseline methods, the Hilbert-augmented algorithms required fewer repeated visits to previously explored cells in order to achieve comparable or greater coverage. Average redundancy decreased for every evaluated team size, with H-PPO consistently producing the lowest redundancy values among all reinforcement learning methods.

**TABLE 3:** Average redundancy (visits per unique cell) across agent team sizes.

Method	4 Agents	8 Agents	12 Agents	16 Agents
DQN	3.477	2.964	3.142	4.173
H-DQN (ours)	**2.395**	**2.184**	**2.071**	**1.948**
PPO	2.962	2.816	2.742	3.354
H-PPO (ours)	**1.985**	**1.891**	**1.841**	**1.768**

The reduction in redundancy was accompanied by improved cumulative coverage, indicating that the additional exploration guidance did not simply reduce robot motion but instead increased exploration efficiency. Representative coverage trajectories generated by the Hilbert-augmented policies exhibited smoother traversal patterns and fewer revisits than those produced by the baseline reinforcement learning algorithms.

Figure 4 illustrates the average cumulative reward obtained throughout training. Both Hilbert-augmented methods demonstrated faster reward accumulation than their corresponding baselines, with the performance gap becoming progressively larger as the number of robots increased. The higher cumulative rewards are consistent with the improvements observed in coverage ratio and redundancy.

**FIGURE 4:**
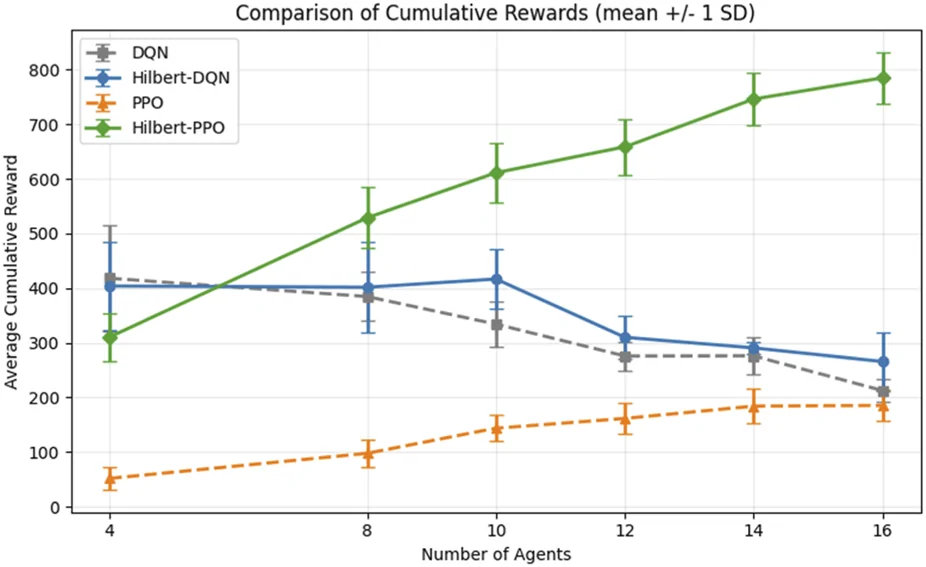
Average cumulative reward across different agent counts for DQN, H-DQN, PPO, and H-PPO. Hilbert-augmented agents consistently achieve higher reward, particularly as the team size increases.

Across all experimental configurations, Hilbert-guided structured exploration produced higher coverage ratios, lower redundancy, and greater cumulative reward than the corresponding baseline reinforcement learning methods. Mean values together with standard deviations and 95% confidence intervals are reported in Tables 2 and 3. Statistical comparisons using Welch’s two-sample t -test indicated that the observed improvements were statistically significant ( p<0.05 ) for all reported coverage and redundancy measurements.

### 3.2 Learning performance

In addition to improving final coverage performance, the proposed framework was designed to increase learning efficiency by encouraging structured exploration during the early stages of reinforcement learning. Learning performance was therefore evaluated using cumulative reward trajectories together with the convergence criterion defined in Section 2.8. Figure 4 presents the average cumulative reward obtained throughout training for all evaluated reinforcement learning algorithms. Each curve represents the mean performance across five independent training runs, while the shaded regions indicate one standard deviation.

For both DQN and PPO, the Hilbert-augmented variants accumulated reward more rapidly than their corresponding baseline algorithms. The difference between the learning curves became apparent during the initial stages of training and remained consistent throughout the optimization process. Among all evaluated methods, H-PPO achieved the highest cumulative reward while exhibiting the smallest variability across independent runs.

Convergence performance was evaluated using the stability criterion described in Section 2.8. To more robustly estimate convergence speed, we use a windowed stability-based metric. Specifically, we track the rolling average reward over a fixed window of episodes and mark convergence when this average remains above 90% of the maximum observed reward for at least 10 consecutive windows. This approach avoids being misled by transient spikes or noise in the reward trajectory and provides a more stable indicator of learning progress. We define $T_{\text{conv}}$ as the first episode where the stability condition holds and average it over five seeds, with 95% CIs from a t -interval, evaluating every 10,000 steps; if the threshold is never met, we record $T_{\text{conv}}=\infty$ for that seed. The observed reduction in convergence time was accompanied by lower variance between independent training runs. In particular, H-PPO exhibited the smallest standard deviation in cumulative reward during the later stages of training, indicating more consistent learning across different random initializations.

The improved learning efficiency was also reflected in sample efficiency. Hilbert-guided exploration enabled both reinforcement learning algorithms to obtain higher cumulative reward using fewer environmental interactions during the early stages of training. Consequently, fewer training episodes were required before stable coverage policies emerged.

### 3.3 Scalability analysis

To evaluate the scalability of the proposed framework, experiments were conducted using robot teams consisting of 4, 8, 12, and 16 agents. Scalability was assessed by examining changes in coverage ratio, redundancy, and cumulative reward as the number of robots increased while maintaining identical training conditions and environment configurations.

Figure 5 compares Hilbert-DQN with a standard DQN baseline across reward, coverage, and redundancy. Hilbert-DQN exhibits steadily increasing cumulative reward as agent count grows, while standard DQN peaks and then collapses in performance at 16 agents. The coverage ratio and redundancy curves reinforce this: Hilbert-DQN achieves broader spatial reach with minimal revisits, whereas standard DQN agents repeatedly visit already-covered areas.

**FIGURE 5:**
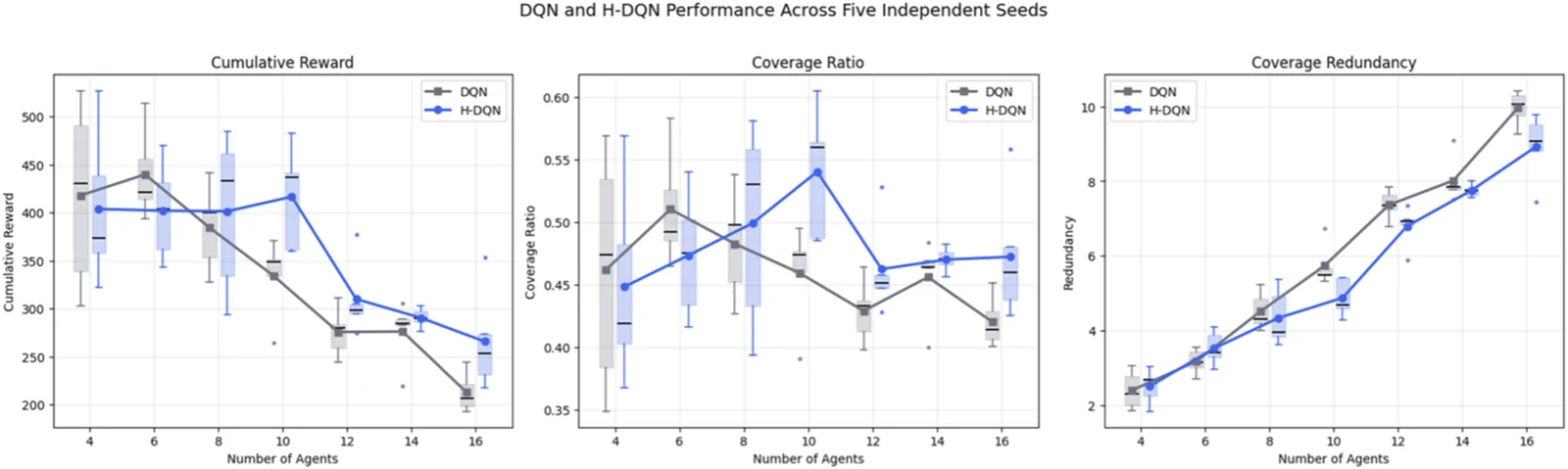
Comparison between H-DQN and standard DQN across cumulative reward, coverage ratio, and redundancy. Hilbert-DQN shows superior scalability and efficiency, especially at higher agent counts.

The Hilbert-PPO scalability results are discussed along with ablation studies in Section 3.4. H-PPO consistently achieves the lowest redundancy, particularly as the number of agents increases. In contrast, standard PPO show increasing redundancy at larger scales, reflecting a lack of coordinated spatial planning.

Although experiments were limited to teams of sixteen robots, the observed trends suggest that the benefits of Hilbert-guided exploration should persist for moderately larger decentralized teams. As team size increases, however, communication constraints, spatial congestion, collision avoidance, and diminishing exploration gains may become increasingly significant. Evaluating the framework in larger robot teams represents an important direction for future work.

### 3.4 Ablation study

To quantify the contribution of each component of the proposed framework, a series of ablation experiments was conducted using the PPO implementation. Three modified versions of the proposed method were evaluated: (i) removal of Hilbert-based state augmentation while retaining curve-guided exploration and reward shaping, (ii) removal of curve-guided exploration while retaining state augmentation and reward shaping, and (iii) removal of reward shaping while preserving the remaining components. Standard PPO, which excludes all three modifications, was also evaluated for comparison.

All ablation experiments were performed using the same network architecture, hyperparameter settings, training duration, and random initialization seeds as the complete H-PPO framework. Performance was evaluated using cumulative reward, coverage ratio, and redundancy.

Figure 6 illustrates the cumulative reward obtained during training for the complete framework and each ablated variant. The complete H-PPO framework consistently accumulated reward more rapidly throughout training than any of the modified variants. The largest separation was observed between the complete framework and the model without curve-guided exploration.

**FIGURE 6:**
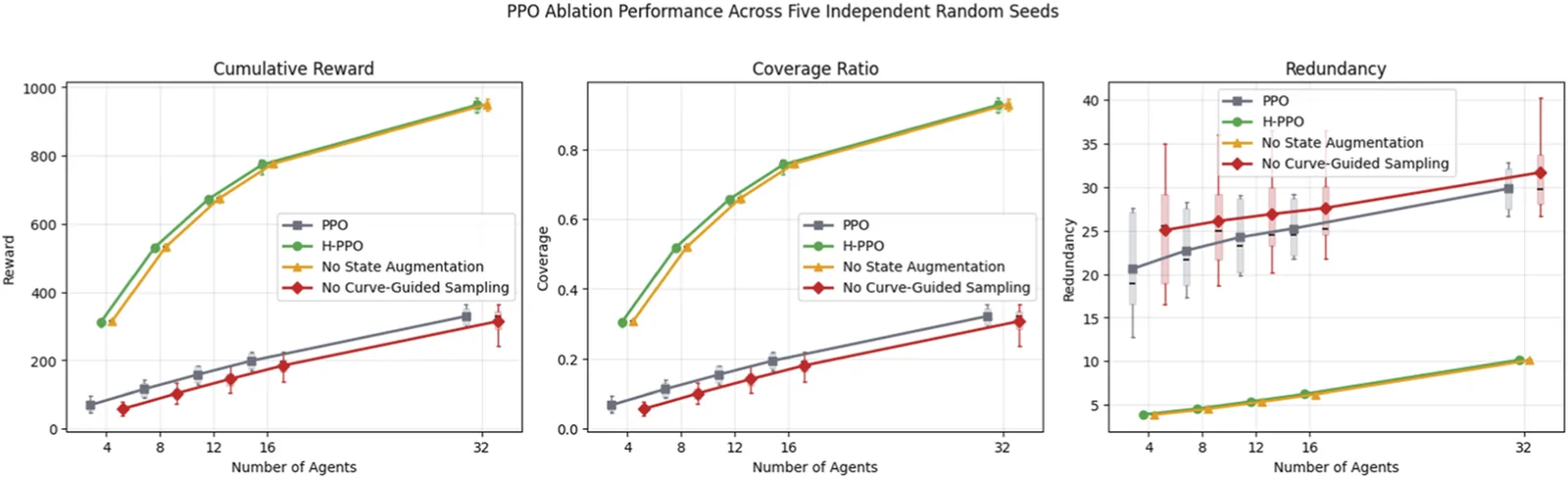
Comparison between PPO and H-PPO. H-PPO maintains low redundancy due to better coverage coordination, while ablated variants show increased overlap.

Figure 6 presents the corresponding coverage ratios obtained during evaluation. Similar trends were observed across all experimental repetitions, with the complete framework consistently producing the highest coverage and the lowest redundancy.

Figure 6 reports the average redundancy, defined as the mean number of visits per unique cell, across different team sizes. Lower values indicate more efficient coverage with reduced overlap.

Removal of Hilbert-based state augmentation produced only a small change in overall performance. The resulting policy achieved coverage and redundancy values comparable to those obtained using the complete H-PPO framework, with differences remaining within one standard deviation across repeated training runs. Similar trends were observed for cumulative reward.

In contrast, removing the curve-guided exploration component resulted in a substantial reduction in performance. Coverage ratio decreased to levels comparable with those observed for the baseline PPO implementation, while redundancy increased considerably. Cumulative reward likewise exhibited a marked reduction throughout the training process.

The reward-shaping ablation produced intermediate performance between the complete framework and the baseline PPO implementation. Although coverage performance remained higher than that achieved by standard PPO, both cumulative reward and convergence speed were reduced relative to the complete H-PPO framework.

The differing impact of the ablated components reflects their distinct roles during policy learning. Hilbert-guided exploration directly influences the trajectories collected throughout training and therefore determines the quality and diversity of the experiences available to the reinforcement learning algorithm. Consequently, removing the exploration bias substantially reduces coverage efficiency and slows policy convergence. In contrast, Hilbert-based state augmentation provides additional global spatial context without directly altering exploration behavior, explaining its comparatively smaller effect on overall performance. Reward shaping occupies an intermediate role by providing denser learning signals that accelerate policy optimization while preserving the optimal policy. Together, these results indicate that the three components are complementary, with exploration guidance serving as the primary mechanism for improving learning efficiency and the remaining components enhancing learning stability and convergence.

### 3.5 Physical validation

To evaluate the transferability of the proposed framework from simulation to real-world deployment, reinforcement learning policies trained using Hilbert-guided exploration were executed on two Boston Dynamics Spot quadruped robots operating in an outdoor environment representative of ecological monitoring missions. The experimental site contained natural terrain features including trees, shrubs, bushes, uneven ground, and other vegetation that constrained robot motion and required adaptive navigation. The objective of these experiments was to determine whether policies learned in simulation could be transferred successfully to a physical robotic platform while maintaining efficient survey behavior under realistic outdoor conditions.

The experimental site was partitioned into discrete survey regions to enable systematic evaluation of exploration performance while preserving the natural layout of the environment. Policy-generated waypoints were converted into executable $SE\left(2\right)$ trajectories using the waypoint interface described in Section 2.6. The learned policies were executed concurrently by the two robots, allowing evaluation of decentralized exploration behavior under realistic outdoor conditions.

Representative ecological survey trajectories are shown in Figure 3. The outdoor environment contains natural obstacles including trees, bushes, and vegetation that interrupt direct traversal and require the robot to adapt its motion while maintaining systematic coverage. Compared with the baseline PPO policy, the proposed H-PPO framework generated smoother locality-preserving trajectories with fewer redundant revisits and more efficient traversal of the survey area. These characteristics are particularly desirable for ecological monitoring, where repeated visits to previously surveyed locations reduce operational efficiency and increase mission duration.

Quantitative hardware performance was evaluated using coverage time, redundancy, and path fidelity. Execution statistics in Table 4 confirm that H-PPO completed field trials 31.9% faster with a 51.6% reduction in redundancy compared to standard PPO. Furthermore, H-PPO achieved a 100% trial success rate and improved coverage efficiency to 94.1 while reducing path-following error. Mean lateral tracking error remained small throughout all experiments, indicating close agreement between the planned trajectories and those recorded from onboard odometry during execution despite the presence of natural environmental obstacles.

**TABLE 4:** Quantitative hardware execution statistics across outdoor field trials (mean ± 1 SD). Standard PPO metrics are averaged over the two completed trials (*N* = 2); H-PPO over three (*N* = 3). Trial success rate is computed over all three attempts.

Metric	Standard PPO	H-PPO	Improvement
Mission duration (s)	512.3 ± 24.1	348.7 ± 11.2	31.9% faster
Coverage efficiency (%)	76.4 ± 4.2	94.1 ± 1.8	+17.7 pp
Redundancy (visits/cell)	2.15 ± 0.18	1.04 ± 0.03	51.6% lower
Path-following error (m)	0.19 ± 0.04	0.08 ± 0.02	57.8% lower
Trial success rate	66.7% (2/3)	100% (3/3)	+33.3 pp

No failures attributable to the Hilbert-guided exploration framework were observed during field deployment. Minor localization drift and heading deviations occurred occasionally during extended traversals; however, these effects were mitigated through waypoint replanning, discrete heading correction, and the trajectory generation procedure described in Section 2.6. Emergency stop protection, lease management, and communication synchronization provided by the Spot SDK remained stable throughout all experimental trials.

The outdoor multi-robot experiments demonstrate that policies learned using Hilbert-guided exploration can be transferred successfully from simulation to a physical robotic platform operating in representative ecological environments. Although the experiments focused on autonomous coverage rather than application-specific sensing, the framework is independent of sensing modality and can be integrated with RGB cameras, multispectral imagers, thermal cameras, LiDAR, hyperspectral sensors, or environmental sensing payloads used for habitat mapping, vegetation surveys, biodiversity assessment, ecosystem restoration, and long-term ecological monitoring.

### 3.6 Summary of findings

The experimental evaluation presented in this section demonstrates consistent improvements in reinforcement learning-based coverage performance through the incorporation of Hilbert-guided structured exploration. Across all evaluated simulation environments and robot team sizes, both H-DQN and H-PPO achieved higher coverage ratios and lower redundancy than their corresponding baseline reinforcement learning algorithms.

Learning performance analysis further showed that the Hilbert-augmented methods accumulated reward more rapidly during training and satisfied the convergence criterion using fewer interaction steps than conventional DQN and PPO. These improvements were consistently observed across five independent training runs, with reduced variability during the later stages of policy optimization.

Scalability experiments demonstrated that the proposed framework maintained its performance advantage as the number of robots increased from four to sixteen. The Hilbert-augmented methods consistently produced higher coverage and lower redundancy across all evaluated team sizes without introducing measurable degradation in learning stability.

The ablation experiments quantified the relative contribution of the proposed framework components. Removal of curve-guided exploration produced the largest reduction in performance, while removal of Hilbert-based state augmentation resulted in comparatively smaller changes. The complete H-PPO framework consistently achieved the highest cumulative reward and coverage ratio together with the lowest redundancy among the evaluated variants.

Finally, the physical experiments demonstrated that policies learned using Hilbert-guided structured exploration can be transferred successfully from simulation to two physical Boston Dynamics Spot robots. The generated waypoint trajectories were executed reliably, exhibiting accurate trajectory tracking and efficient coverage behavior under repeated experimental trials.

## 4 Discussion

### 4.1 Interpretation of the experimental findings

The primary objective of this study was to investigate whether incorporating locality-preserving geometric priors into reinforcement learning improves exploration efficiency for decentralized multi-robot coverage. The experimental results presented in Section 3 consistently support this hypothesis. Across all evaluated environments, Hilbert-guided structured exploration produced higher coverage ratios, lower redundancy, faster convergence, and greater cumulative reward than the corresponding baseline reinforcement learning algorithms. These improvements were observed for both value-based (H-DQN) and policy-gradient (H-PPO) methods, indicating that the proposed framework is largely independent of the underlying reinforcement learning algorithm.

One of the most consistent findings throughout the experimental evaluation was the reduction in redundant cell visitation. Coverage tasks are particularly sensitive to inefficient exploration because repeated visits to previously explored regions reduce the amount of useful information obtained from each interaction with the environment. The Hilbert-guided exploration strategy consistently reduced redundancy while simultaneously increasing the proportion of the workspace visited during each episode. These observations indicate that introducing geometric structure into the exploration process enables reinforcement learning agents to acquire more informative experiences than conventional stochastic exploration alone.

The observed improvements can be explained by the locality-preserving properties of the Hilbert space-filling curve. Consecutive Hilbert indices correspond, whenever possible, to neighboring cells in the workspace, naturally encouraging smooth and spatially coherent exploration trajectories. During the early stages of reinforcement learning, when policy estimates remain highly uncertain, standard exploration strategies frequently generate fragmented trajectories that repeatedly revisit previously explored regions while failing to discover unexplored areas. Hilbert-guided exploration reduces this inefficiency by providing a structured exploration bias that encourages systematic traversal without constraining the learned policy to follow a predetermined path. As the exploration probability is gradually annealed, decision making increasingly reflects the learned policy rather than the geometric prior, preserving the adaptability that characterizes reinforcement learning.

The learning performance results further support this interpretation. Both Hilbert-augmented methods accumulated reward more rapidly and satisfied the convergence criterion using fewer interaction steps than their corresponding baseline implementations. Faster convergence suggests that structured exploration improves sample efficiency by increasing the proportion of informative state transitions encountered during training. Rather than modifying the optimization procedures of DQN or PPO, the proposed framework improves the quality of the training data from which these algorithms learn.

The scalability experiments demonstrate that these benefits persist as the number of robots increases from four to sixteen. Importantly, higher coverage ratios and lower redundancy were maintained without introducing explicit communication, shared maps, or centralized coordination. Although the experiments do not demonstrate explicit cooperative planning, they indicate that structured exploration alone can substantially reduce overlap among independently operating robots. This observation suggests that geometric priors provide an effective means of improving decentralized coverage efficiency while maintaining the simplicity of independent reinforcement learning agents.

The ablation study provides additional insight into the mechanisms responsible for the observed improvements. Among the evaluated variants, removal of curve-guided exploration produced the largest decrease in coverage ratio, cumulative reward, and convergence speed. In contrast, removal of Hilbert-based state augmentation resulted in comparatively small changes in overall coverage performance. These findings indicate that the principal benefit of the proposed framework arises from structured exploration rather than from the inclusion of the Hilbert index as an additional state feature. Nevertheless, the reduced variability observed across repeated training runs suggests that state augmentation contributes to learning stability, particularly during later stages of optimization.

The differing influence of the three components can be explained by their distinct roles during reinforcement learning. Hilbert-guided exploration directly determines the trajectories collected during training, thereby influencing both the diversity and informativeness of the experiences stored for policy optimization. By encouraging systematic visitation of previously unexplored regions while reducing redundant revisits, the exploration bias improves sample efficiency and has the greatest impact on overall learning performance. In contrast, Hilbert-based state augmentation contributes only a single normalized spatial feature that provides global positional context without directly altering robot behavior. Its primary benefit is therefore to reduce state ambiguity and improve policy stability, particularly during the later stages of training when the policy begins to exploit previously acquired knowledge. Potential-based reward shaping occupies an intermediate role by providing denser learning signals in sparse-reward environments, allowing the agent to associate local actions with long-term exploration progress more rapidly while preserving the optimal policy. Collectively, these mechanisms are complementary: exploration guidance improves the quality of collected experience, state augmentation enhances representation and policy stability, and reward shaping accelerates convergence by providing more informative training feedback.

An important characteristic of the proposed framework is that the Hilbert curve functions as an exploration scaffold rather than as a deterministic coverage planner. Classical coverage path planning algorithms prescribe complete trajectories that robots are expected to follow during execution. In contrast, the proposed framework influences only the exploration process during learning. Once an effective policy has been acquired, robot actions are determined primarily by the reinforcement learning policy itself rather than by the Hilbert ordering. Consequently, the proposed approach combines the spatial coherence associated with deterministic coverage planning with the adaptability of reinforcement learning.

The successful deployment of the learned policies on two Boston Dynamics Spot robots further demonstrates that the proposed framework is not restricted to simulation. Policies trained using Hilbert-guided structured exploration were transferred successfully to a physical robotic platform and executed through the proposed waypoint interface. The successful deployment on two Boston Dynamics Spot robots demonstrates that the proposed framework extends beyond simulation and can support decentralized multi-robot exploration in representative outdoor environments.

### 4.2 Comparison with existing methods

The proposed framework occupies an intermediate position between classical coverage path planning and reinforcement learning-based exploration. Classical coverage algorithms, including boustrophedon decomposition, spiral traversal, and Hilbert curve planning, generate deterministic trajectories that provide highly efficient coverage (Jayalakshmi et al., 2025; Kapoutsis et al., 2017; Kusnur and Likhachev, 2022) in structured environments where complete knowledge of the workspace is available. Their computational efficiency and predictable behavior have made them widely used for applications such as robotic inspection, floor cleaning, and environmental surveying. However, these approaches generally assume that the environment is known *a priori* and require replanning when obstacles or environmental conditions change.

In contrast, reinforcement learning learns navigation policies directly through interaction with the environment. This adaptability allows learned policies to respond to previously unseen situations without explicitly recomputing coverage trajectories. The trade-off is that reinforcement learning often requires substantially greater exploration during training, particularly in sparse-reward environments where informative experiences are difficult to obtain. The present work addresses this limitation by introducing a geometric prior that guides exploration without replacing the learning process itself.

Several previous studies have sought to improve exploration efficiency through intrinsic motivation, curiosity-driven learning, episodic memory, curriculum learning, or map-based exploration (Gupta et al., 2018; Calzolari et al., 2025; Chiun et al., 2025). These approaches generally increase the amount of information available to the learning algorithm or modify the reward function to encourage exploration of novel states. While effective in many applications, such methods frequently introduce additional optimization objectives, auxiliary neural networks, or memory structures that increase computational complexity and training cost.

The proposed framework differs conceptually from these methods by incorporating geometric knowledge directly into the exploration process. Rather than learning an auxiliary exploration objective, the Hilbert space-filling curve provides a deterministic locality-preserving prior that guides exploration during the early stages of policy learning. Because Hilbert index computation requires only integer arithmetic and introduces a single additional scalar into the observation space, the computational overhead of the proposed method remains negligible relative to the reinforcement learning algorithms themselves.

Another important distinction concerns the role of the geometric prior. In conventional coverage path planning, the space-filling curve defines the trajectory that the robot follows throughout execution. In the present framework, the Hilbert ordering serves only as an exploration scaffold during training. As the exploration probability is gradually annealed, the learned policy increasingly determines robot behavior while retaining the flexibility to deviate from the geometric traversal whenever this improves long-term reward. Consequently, the proposed approach combines the adaptability of reinforcement learning with the spatial coherence associated with classical coverage strategies.

The experimental results also suggest that the proposed framework is largely independent of the underlying reinforcement learning algorithm. Both value-based learning (H-DQN) and policy-gradient learning (H-PPO) exhibited consistent improvements in coverage ratio, redundancy, and convergence speed relative to their respective baseline implementations. This observation indicates that Hilbert-guided structured exploration functions primarily as an exploration mechanism rather than as an algorithm-specific optimization procedure.

The present study focuses on DQN and PPO because they represent two of the most widely used reinforcement learning paradigms for robotic navigation and coverage. More recent multi-agent reinforcement learning algorithms, including MAPPO, MADDPG, and QMIX (Lowe et al., 2017; Yu et al., 2021; Rashid et al., 2018; Foerster et al., 2018), incorporate additional mechanisms for cooperative learning (Zhong et al., 2024; Albrecht et al., 2024; Yu et al., 2023) and centralized training. Although these methods were not evaluated in the present work, the proposed structured exploration framework is algorithmically independent of the underlying optimization procedure and could, in principle, be integrated with other actor–critic or value-based reinforcement learning algorithms. Evaluating the compatibility of Hilbert-guided exploration with modern multi-agent reinforcement learning methods represents an important direction for future research. The proposed framework should therefore be viewed as complementary to curiosity-driven, frontier-based, and cooperative exploration methods rather than as an alternative reinforcement learning algorithm.

### 4.3 Practical implications

The results of this study demonstrate that incorporating lightweight geometric priors into reinforcement learning provides a practical mechanism for improving autonomous ecological monitoring. By guiding exploration through locality-preserving spatial information, the proposed framework increases survey coverage, reduces redundant traversal, and accelerates policy learning without modifying the underlying reinforcement learning algorithms. These characteristics are particularly important for ecological monitoring missions, where robotic platforms must efficiently survey large natural environments while operating under limited energy, communication, and computational resources.

A key advantage of the proposed framework is its computational simplicity. The complexity analysis presented in Section 2.5 further shows that the additional computations introduced by Hilbert-guided exploration are constant-time operations that do not alter the asymptotic complexity of the underlying reinforcement learning algorithms, making the framework suitable for deployment on computationally constrained robotic platforms. Hilbert index computation requires only integer arithmetic and introduces a single additional scalar into the observation space. Unlike approaches based on intrinsic motivation, auxiliary prediction networks, or memory-augmented reinforcement learning, the proposed method does not require additional trainable models or complex optimization objectives. Consequently, the framework can be integrated into existing reinforcement learning systems with minimal modification while introducing negligible computational overhead during both training and deployment.

The reduction in redundant exploration observed throughout the experimental evaluation has direct implications for ecological monitoring missions. Repeated visits to previously surveyed locations increase mission duration, battery consumption, and mechanical wear while reducing the number of unique sampling locations that can be visited during a deployment. In applications such as habitat mapping, vegetation surveys, biodiversity assessment, wetland monitoring, invasive species detection, and ecosystem restoration, efficient exploration directly influences the spatial quality and completeness of the collected environmental data. By reducing unnecessary revisits, the proposed framework enables robotic platforms to allocate a greater proportion of their operational time to acquiring new observations.

The occupancy-grid environments used in this study abstract the geometric structure commonly encountered in natural ecosystems. Traversable cells represent accessible survey locations, while occupied cells correspond to terrain features such as dense vegetation, mature trees, shrubs, rock outcrops, wetlands, streams, and protected habitat zones that constrain robot mobility. Although simplified, this representation captures the fundamental coverage challenges encountered during ecological survey missions while providing a reproducible benchmark for evaluating exploration strategies.

The outdoor experiments conducted using two Boston Dynamics Spot robots further demonstrate the practical feasibility of the proposed framework. Policies trained entirely in simulation were successfully deployed in an outdoor environment containing natural vegetation, trees, bushes, and other terrain features representative of ecological monitoring sites. The resulting survey trajectories preserved the efficient exploration behavior observed during simulation while adapting to realistic environmental constraints, providing evidence that the proposed framework can support autonomous field deployment.

An additional practical advantage is the framework’s independence from sensing modality. The proposed method modifies only the exploration process and is therefore compatible with a wide range of sensing systems, including RGB cameras, multispectral and hyperspectral imagers, thermal cameras, LiDAR, and environmental sensing payloads. Consequently, the framework can support a broad range of ecological applications, including forest inventory, biodiversity monitoring, vegetation health assessment, habitat restoration, wetland assessment, precision conservation, and long-term environmental monitoring.

The framework also operates without explicit inter-robot communication, allowing decentralized robotic teams to perform systematic exploration in large natural environments where communication may be intermittent or unavailable because of terrain, vegetation, or operational distance. This property makes the proposed approach particularly attractive for large-scale ecological monitoring missions spanning forests, grasslands, wetlands, coastal ecosystems, and other environmentally sensitive regions. Thus, the framework proposed in this paper can be integrated with existing ecological sensing pipelines.

Finally, because the proposed framework is algorithmically independent of the underlying reinforcement learning method, it can be incorporated into a broad range of value-based, policy-gradient, and actor–critic algorithms without modifying their optimization procedures. This flexibility broadens the applicability of the approach to future autonomous robotic systems designed for long-duration ecological monitoring and ecosystem restoration.

### 4.4 Limitations

Although the proposed framework demonstrated consistent improvements in coverage efficiency and learning performance across the evaluated experiments, several limitations should be considered when interpreting the results.

First, the proposed framework does not implement explicit inter-robot communication or centralized coordination. Each robot makes decisions independently using locally available observations together with the Hilbert-guided exploration mechanism. Consequently, the reduction in coverage redundancy observed throughout the experiments should not be interpreted as evidence of explicit cooperative planning. Instead, the results indicate that locality-preserving geometric priors can improve decentralized exploration efficiency even in the absence of communication. Extending the framework to communication-aware multi-robot systems represents an important direction for future investigation.

Second, the simulation experiments were conducted using occupancy-grid benchmark environments that abstract the geometric characteristics of ecological survey regions. Although these environments capture fundamental coverage and exploration challenges, they do not explicitly model many factors encountered during ecological monitoring, including heterogeneous terrain traversability, seasonal vegetation changes, weather conditions, wildlife interactions, sensor uncertainty, or continuously varying terrain. Evaluating the proposed framework within high-fidelity ecological simulation environments and larger outdoor field deployments will provide a more comprehensive assessment of its applicability to real-world monitoring missions.

Third, although the outdoor experiments validated decentralized exploration using two Boston Dynamics Spot robots, the evaluation remains limited in scale compared with large multi-robot ecological monitoring missions involving larger robot teams. Although the experimental site contained natural vegetation, trees, bushes, and other terrain features representative of ecological monitoring environments, the experiments primarily validated autonomous exploration and trajectory execution rather than complete ecological survey workflows. The platform was not equipped with application-specific environmental sensing payloads such as multispectral cameras, hyperspectral imagers, thermal sensors, LiDAR, or environmental sampling instruments. Consequently, the present study demonstrates the feasibility of autonomous navigation and coverage but does not evaluate ecological data acquisition or analysis.

A further limitation concerns the range of reinforcement learning algorithms evaluated in this study. The proposed framework was integrated with Deep Q-Networks and Proximal Policy Optimization because these algorithms represent widely adopted value-based and policy-gradient reinforcement learning approaches. More recent multi-agent reinforcement learning methods, including MAPPO, QMIX, and MADDPG, incorporate centralized training and additional mechanisms for cooperative policy learning that were not considered here. Although the proposed framework is algorithmically independent of the underlying optimization procedure, experimental evaluation using these methods would provide a broader assessment of its generality.

The present work also focuses exclusively on Hilbert space-filling curves as the geometric prior. Other locality-preserving spatial representations, including Morton (Z-order) curves, Peano curves, adaptive space-filling curves, and learned spatial embeddings, were not investigated. Comparative evaluation of these alternatives would provide additional insight into the extent to which the observed improvements arise from the specific properties of the Hilbert traversal rather than from geometric priors more generally.

Finally, although statistical analysis was performed using multiple independent training runs, the experiments were conducted within a limited number of simulated environments and one representative outdoor test site. Additional evaluation across diverse ecological settings, including forests, wetlands, grasslands, coastal ecosystems, and restoration sites, together with different terrain characteristics and sensing configurations, would strengthen the empirical conclusions and improve understanding of the conditions under which Hilbert-guided exploration provides the greatest benefit.

Despite these limitations, the proposed framework consistently improved coverage efficiency, learning performance, and deployment feasibility while introducing only negligible computational overhead. The results demonstrate that locality-preserving geometric priors provide a promising foundation for future autonomous ecological monitoring systems and motivate further investigation using richer environmental models, advanced sensing modalities, and large-scale multi-robot field deployments.

### 4.5 Future research directions

The results presented in this study suggest several promising directions for extending the proposed framework. Although Hilbert-guided structured exploration consistently improved reinforcement learning performance across the evaluated coverage tasks, additional research is required to determine its applicability to more complex robotic environments and learning paradigms.

A natural extension of the present work is the evaluation of alternative geometric priors for structured exploration. While Hilbert space-filling curves were selected because of their strong locality-preserving properties, other spatial indexing methods, including Morton (Z-order) curves, Peano curves, and adaptive space-filling curves, may provide different trade-offs between computational efficiency and exploration performance. Comparative studies would improve understanding of the relationship between geometric representation and reinforcement learning efficiency.

Future work should also investigate integration with contemporary multi-agent reinforcement learning algorithms. Recent advances in cooperative reinforcement learning, including MAPPO and QMIX, provide promising directions for extending the proposed framework to explicitly coordinated multi-robot systems (Yu et al., 2021; Rashid et al., 2018; Zhong et al., 2024; Albrecht et al., 2024). The present study focused on DQN and PPO because they represent widely adopted value-based and policy-gradient methods. Extending the proposed framework to algorithms such as MAPPO, QMIX, MADDPG, or other centralized training and decentralized execution approaches would provide a broader assessment of the generality of Hilbert-guided structured exploration and determine whether similar improvements are obtained in explicitly cooperative learning settings.

Another important direction concerns operation in continuous and dynamic environments. The current experiments were conducted using discrete occupancy grids with static environmental structure. Real-world robotic systems frequently operate in continuous workspaces containing moving obstacles, uncertain localization, and incomplete environmental information. Incorporating adaptive geometric priors together with online obstacle avoidance and dynamic replanning mechanisms may improve the robustness of structured exploration under these conditions.

The hardware experiments performed in this study demonstrate the feasibility of transferring learned policies from simulation to physical quadruped robots. An important next step is the deployment of larger heterogeneous robot teams consisting of ground, aerial, and aquatic platforms operating simultaneously in shared environments. Multi-robot hardware experiments would permit evaluation of communication constraints, synchronization, collision avoidance, and cooperative task allocation under realistic operating conditions while providing further validation of the proposed framework.

The present framework may also be extended to heterogeneous robotic teams consisting of aerial, ground, and legged platforms (Wakilpoor et al., 2020; Agarwal et al., 2025). Such systems could exploit complementary sensing modalities and mobility characteristics to perform collaborative coverage of large environments. Investigating structured exploration within heterogeneous robot teams represents an important research direction for applications requiring efficient monitoring of complex three-dimensional environments.

Finally, the computational simplicity of Hilbert-guided structured exploration makes it attractive for long-duration autonomous inspection and monitoring tasks. Future work will investigate the integration of the proposed framework with perception, semantic mapping, and digital-twin technologies to support autonomous inspection of wetlands, forests, coral reefs, coastal ecosystems, reforestation sites, and invasive species monitoring In these applications, reinforcement learning can provide adaptive decision making while structured exploration offers an efficient mechanism for systematically acquiring spatial information over extended operational periods.

## 5 Conclusion

This study investigated the use of locality-preserving geometric priors to improve reinforcement learning for decentralized multi-robot ecological monitoring. The motivation for this work was the observation that reinforcement learning often suffers from inefficient exploration and slow policy convergence in large-scale coverage tasks, while classical coverage path planning algorithms generally lack the adaptability required for dynamic or partially known natural environments. To address these challenges, we introduced a lightweight framework that incorporates Hilbert space-filling curve priors through state augmentation, exploration biasing, and potential-based reward shaping while preserving the underlying reinforcement learning algorithms.

The proposed framework was evaluated using both value-based and policy-gradient reinforcement learning methods. Extensive simulation experiments demonstrated consistent improvements in coverage ratio, redundancy, cumulative reward, and convergence speed relative to conventional DQN and PPO implementations. These improvements were maintained across robot teams ranging from four to sixteen agents, indicating that the proposed approach remains effective as the scale of the monitoring task increases. Furthermore, the ablation study demonstrated that Hilbert-guided exploration constitutes the primary source of the observed performance gains, while Hilbert-based state augmentation contributes primarily to learning stability and reduced performance variability.

The framework was further validated through outdoor experiments using two Boston Dynamics Spot quadruped robots operating in an environment containing natural vegetation, trees, bushes, and other terrain features representative of ecological monitoring sites. Policies trained entirely in simulation were successfully transferred to the physical platform and executed through the proposed waypoint interface, demonstrating reliable trajectory execution and systematic exploration under realistic environmental conditions. Although the hardware experiments focused on autonomous navigation and coverage rather than ecological sensing, they demonstrate that the proposed framework can be integrated with robotic systems intended for field deployment.

The principal contribution of this work is not the introduction of a new reinforcement learning algorithm, but rather the demonstration that lightweight locality-preserving geometric priors can substantially improve the exploration process that underlies reinforcement learning. By combining the systematic spatial organization of Hilbert space-filling curves with the adaptability of reinforcement learning, the proposed framework provides a general exploration methodology that is computationally efficient, algorithmically independent, and readily transferable across different reinforcement learning algorithms and robotic platforms.

From the perspective of ecological robotics, the proposed framework provides a practical foundation for autonomous systems that must repeatedly survey large natural environments while minimizing redundant traversal and operating under limited communication. Because the method is independent of sensing modality, it can be integrated with RGB cameras, multispectral and hyperspectral imagers, thermal cameras, LiDAR, and environmental sensing payloads for applications including habitat mapping, vegetation monitoring, biodiversity assessment, ecosystem restoration, invasive species detection, and long-term environmental observation.

## Data Availability

The original contributions presented in the study are included in the article/supplementary material, further inquiries can be directed to the corresponding author.
